# *STOML1* as an Exploratory Candidate Gene for Autosomal Dominant Iris and Chorioretinal Coloboma in a British Family

**DOI:** 10.3390/genes17091152

**Published:** 2026-09-20

**Authors:** Vanita Berry, Manav B. Ponnekanti, Nancy Aychoua, Maddy Ashwin Reddy, Michel Michaelides

**Affiliations:** 1 UCL Institute of Ophthalmology, University College London, 11-43 Bath Street, London EC1V 9EL, UK; nancy.aychoua@nhs.net; 2 Moorfields Eye Hospital NHS Foundation Trust, London EC1V 2PD, UK; ashwin.reddy4@nhs.net; 3 UCL Medical School, University College London, 74 Huntley St, London WC1E 6DE, UK; m.ponnekanti@ucl.ac.uk; 4 Oxford Eye Hospital NHS Foundation Trust, John Radcliffe Hospital, Headley Way, Oxford OX3 9DU, UK; 5 Department of Ophthalmology, Royal London Hospital, Barts Health NHS Trust, Whitechapel Road, London E1 1FR, UK

**Keywords:** ocular coloboma, autosomal dominant, whole exome sequencing

## Abstract

Background: Ocular coloboma is a congenital eye defect with high genetic heterogeneity. This study investigated a four-generation pedigree to identify candidate variants underlying autosomal dominant iris and chorioretinal coloboma. Methods: Whole-exome sequencing (WES) was performed on a single affected family member. Variants were prioritised using the Phenopolis pipeline, filtered for rarity across multiple population databases, and retained where CADD indicated predicted deleteriousness. Candidate variants were then validated by Sanger sequencing in the four family members from whom DNA was available. Variants were classified according to ACMG/AMP criteria. Results: Five rare heterozygous variants were identified: *STOML1* (NM_004809.5:c.1100T>C; p.(Leu367Pro)), *MTIF2* (NM_002453.3:c.1337G>A; p.(Trp446Ter)), *CDH23* (NM_022124.6:c.8906G>A; p.(Arg2969His)), *CDON* (NM_001378964.1:c.3276+1G>T; p.?), and *ERCC6L2* (NM_020207.7:c.19C>T; p.(Gln7Ter)). Four are classified as of uncertain significance; the *ERCC6L2* variant is classified as pathogenic in ClinVar. Conclusions: *STOML1* is proposed as an exploratory candidate gene for isolated ocular coloboma, requiring replication in independent families and functional validation. The recurrent *ERCC6L2* nonsense variant is reported as an incidental finding of potential haematological relevance.

## 1. Introduction

Ocular coloboma is a congenital, genetically heterogeneous eye defect caused by the optic fissure failing to close during gestation, potentially impacting the iris, lens, zonules, ciliary body, choroid, retina, and optic disc. Globally, coloboma is responsible for 3.2% to 11.2% of childhood blindness and 5% to 10% among children of European ancestry [1,2]. Coloboma tends to be inherited as an autosomal dominant trait but may also occur sporadically or as part of a syndrome such as Coloboma, Heart defects, Atresia of the choanae, Retardation of growth and development, Genital abnormalities, and Ear abnormalities (CHARGE) or Cerebellar vermis hypoplasia, Oligophrenia, Ataxia, Coloboma, and Hepatic fibrosis (COACH) syndrome [3]. Coloboma is most often studied within the wider microphthalmia, anophthalmia, and coloboma (MAC) spectrum and less frequently as an isolated condition [4,5,6]. Sporadic coloboma is common and may result from environmental disruptors [7].

Several studies highlight a complex genetic landscape where localised developmental disruptions or systemic genetic disorders cause the condition. To date, research has linked more than eighty genes to MAC [8,9,10]. Transcription factors represent the largest group of these genes, including *PAX6*, *PITX3*, *SOX2*, *RAX*, *SALL2*, *SIX6*, *VSX2*, *OTX2*, *FOXE3*, and *ATOH7*. Other identified genetic contributors include TGF-β and BMP signalling molecules (*BMP2*, *BMP4*, *BMP7*, *GDF3*, and *GDF6*) as well as components of the retinoic acid pathway (*STRA6*, *RARB*, and *ALDH1A3*) and the Hippo signalling pathway and associated transcriptional regulators (*FAT1*, *RERE*, and *YAP1*). Multiple additional genes, including *SHH*, *MAB21L2*, *TENM3*, *MAF*, *BMPR1B*, *ABCB6*, *DPYD*, *CRIM1*, *FBN1*, *NTN1*, and *TWF1*, are linked to MAC [11]. However, the genetic origin of many non-syndromic MAC cases remains unresolved, highlighting the need for further genetic investigation [12].

## 2. Materials and Methods

**Figure 1:**
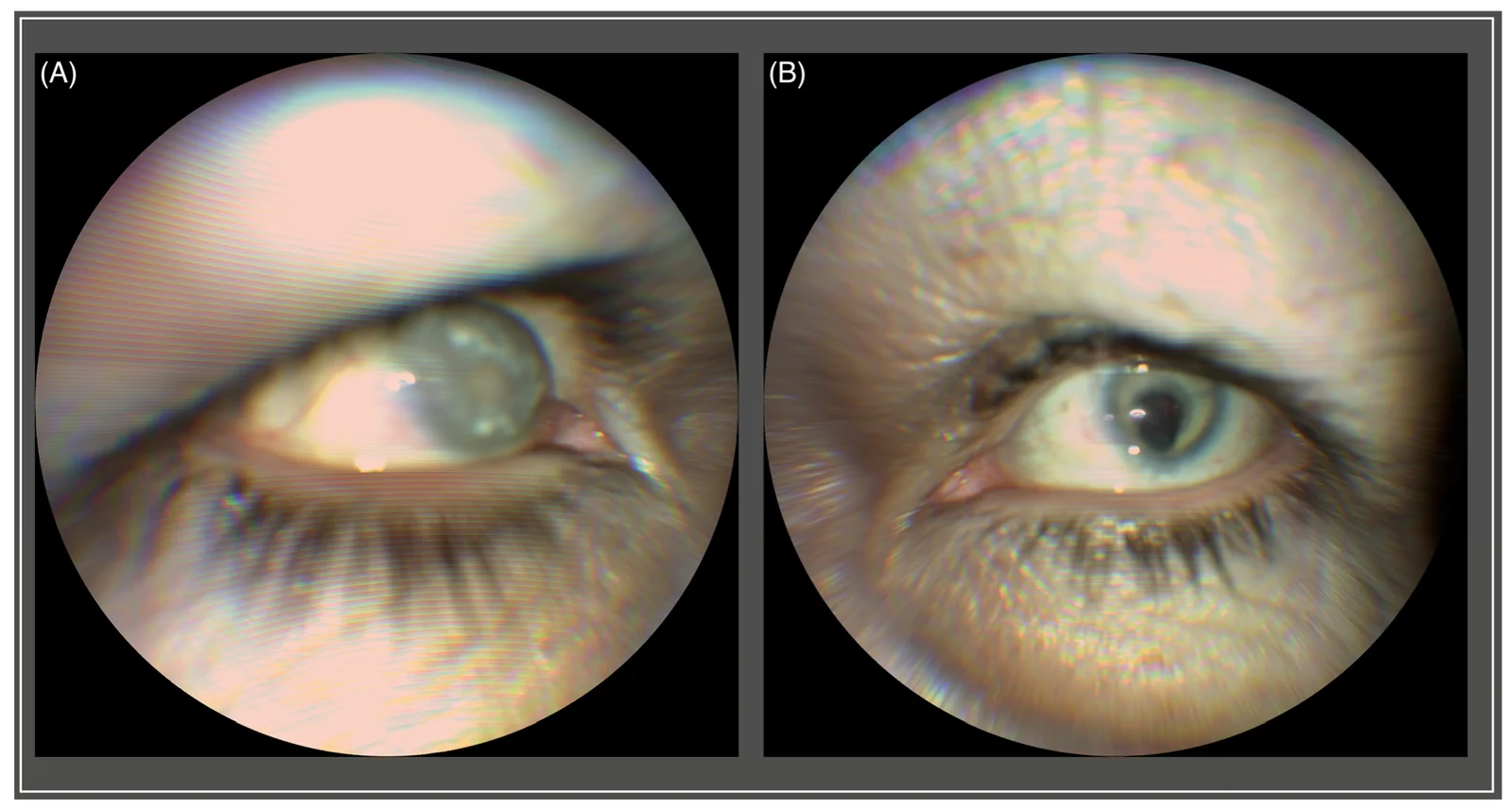
Anterior segment photographs of the proband (III-6) demonstrating bilateral inferonasal iris coloboma. (**A**) The right eye (OD) showing a dense cataract and inferonasal iris coloboma. (**B**) The left eye (OS) demonstrating an inferonasal iris coloboma with a posterior chamber intraocular lens following cataract surgery. The apparent upward gaze of the right eye is due to nystagmus.

**Figure 2:**
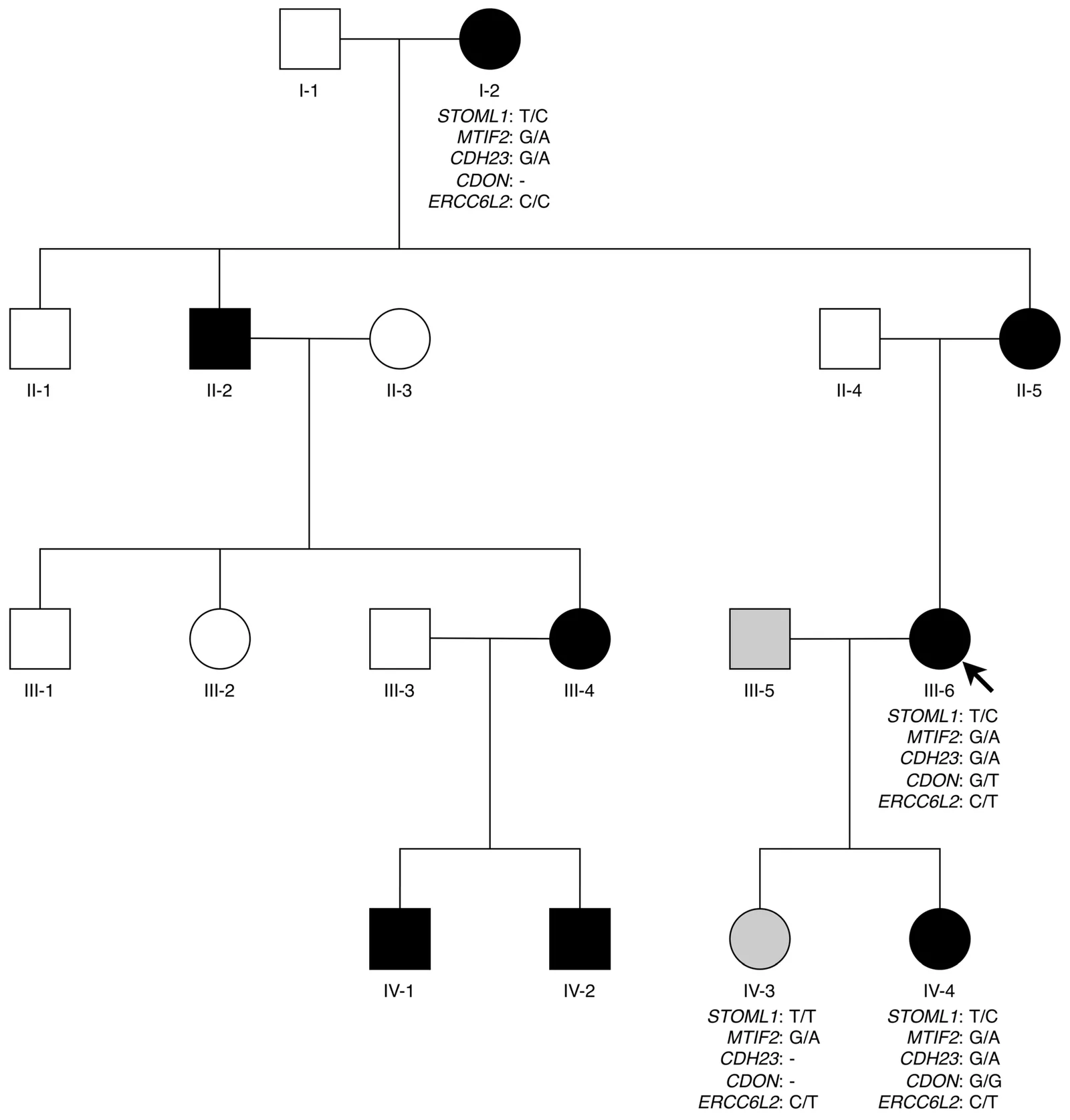
The abridged pedigree of the family. Squares represent males and circles represent females; the arrow indicates the proband (III-6). Filled black symbols indicate ocular coloboma and filled grey symbols indicate cataract without coloboma. Open symbols indicate individuals reported as clinically unaffected; of these, only those examined at Moorfields Eye Hospital were formally assessed. Genotypes are shown for the four individuals from whom DNA was available (I-2, III-6, IV-3, and IV-4); dashes (-) indicate assays that were not performed or that failed. No DNA was available from any individual unaffected by coloboma other than IV-3. The cataract in IV-3 was inherited from her father (III-5); the cataract in III-6 is separate and acquired. Three individuals for whom no phenotypic data were available are omitted from the figure.

The patients were seen at the Genetic Service, Moorfields Eye Hospital, London, UK. The study protocol adhered to the conventions of the Declaration of Helsinki and received approval from the UCL Research Ethics Committee (project ID: 4818.001). Following the provision of written informed consent, all participants were subjected to a full ophthalmic examination, which led to affected individuals being diagnosed with ocular coloboma (Figure 1 and Figure 2).

### 2.1. Clinical Details of the Family

In general, the family has a history of coloboma affecting the iris and retina, with varying severity. The proband (III-6), aged sixty at her most recent examination, has congenital bilateral iris and chorioretinal coloboma, nystagmus, and cataract that developed during her first pregnancy (Figure 1). At the most recent examination, best-corrected visual acuity was hand movements in the right eye and 6/60 in the left eye. Her husband (III-5) has congenital cataracts, bilateral aphakia, and nystagmus. Cataract extraction was performed at 36 years of age and was subsequently complicated by retained lens material requiring pars plana vitrectomy. He also has hypercholesterolaemia, gout, osteoarthritis, and an acquired heart murmur. The proband’s first daughter (IV-3) has congenital cataracts inherited from her father, which were surgically treated at three years of age. She has no evidence of coloboma and is otherwise in good health. The proband’s second daughter (IV-4) has unilateral iris and chorioretinal coloboma; the fellow eye is structurally normal. The affected eye is fitted with a cosmetic shell. Her visual impairment has affected her educational attainment; she also has anxiety and polycystic ovary syndrome. Within the family, the proband (III-6) is the most severely affected, followed by her grandmother (I-2). In contrast, her mother (II-5) had a milder phenotype and retained sufficient vision to drive (Figure 2).

### 2.2. Whole Exome Sequencing and Bioinformatics

Genomic DNA was extracted from EDTA-treated blood using the Nucleon II DNA Extraction Kit (Scotlab Bioscience, Strathclyde, Glasgow, UK). Exome capture and enrichment used the SureSelectXT Human All Exon V6 kit (Agilent Technologies, Santa Rosa, CA, USA), and paired-end sequencing was performed on an Illumina HiSeq 2500 (Macrogen Europe, Amsterdam, The Netherlands) to a mean depth of 50×. The reads were aligned to the GRCh37/hg19 reference assembly and analysed through the Phenopolis pipeline [13]. Genes previously associated with coloboma were examined first using the available WES variant calls. SNVs and small indels were assessed, including coding and splice-associated variants, and were annotated for population frequency, predicted functional consequence, and computational pathogenicity. No previously reported pathogenic SNV or small indel explaining the phenotype was identified among these variant calls. Only the proband (III-6) underwent WES, whilst the other three relatives underwent targeted Sanger sequencing. An amount of 104,699 of 120,922 retained variant calls (86.6%) carried a PASS filter flag; the median read depth at called coding and splice-site positions was 100×, with 95.0% at ≥20×. The transition/transversion ratio was 2.93 across coding single-nucleotide calls and 2.30 across all retained single-nucleotide calls. Heterozygous calls accounted for 0.59 of X-chromosome calls and 0.61 of autosomal calls, and no Y-chromosome calls were present, consistent with the sex of the proband.

A tier-based approach was used for variant prioritisation. Coding variants were retained only if rare in the Genome Aggregation Database (gnomAD) v4.1.0; https://gnomad.broadinstitute.org/ (last accessed on 21 August 2026), defined as an allele frequency below 0.001. Filtering was performed first within known coloboma-associated genes and subsequently extended genome-wide. Subsequently, variants were filtered on combined annotation dependent depletion (CADD) score, retaining those predicted to be moderately or highly deleterious (CADD > 15). This identified 126 rare coding or splice-site variants. The five reported here were selected from that set on allele frequency across the population databases interrogated, in which *MTIF2*, *STOML1*, and *CDH23* rank first, sixth, and thirteenth of the 126, together with relevance of the gene to ocular development or to an established disease association; *CDON* was retained from the first-pass screen of known coloboma-associated genes and *ERCC6L2* as a recurrent variant classified as pathogenic in ClinVar. Further annotation of these variants was performed using VarSome version 11.9; https://varsome.com (last accessed on 15 May 2026), and variants were classified according to the ACMG/AMP guidelines [14] (Table 1).

### 2.3. Sanger Sequencing

**Figure 3:**
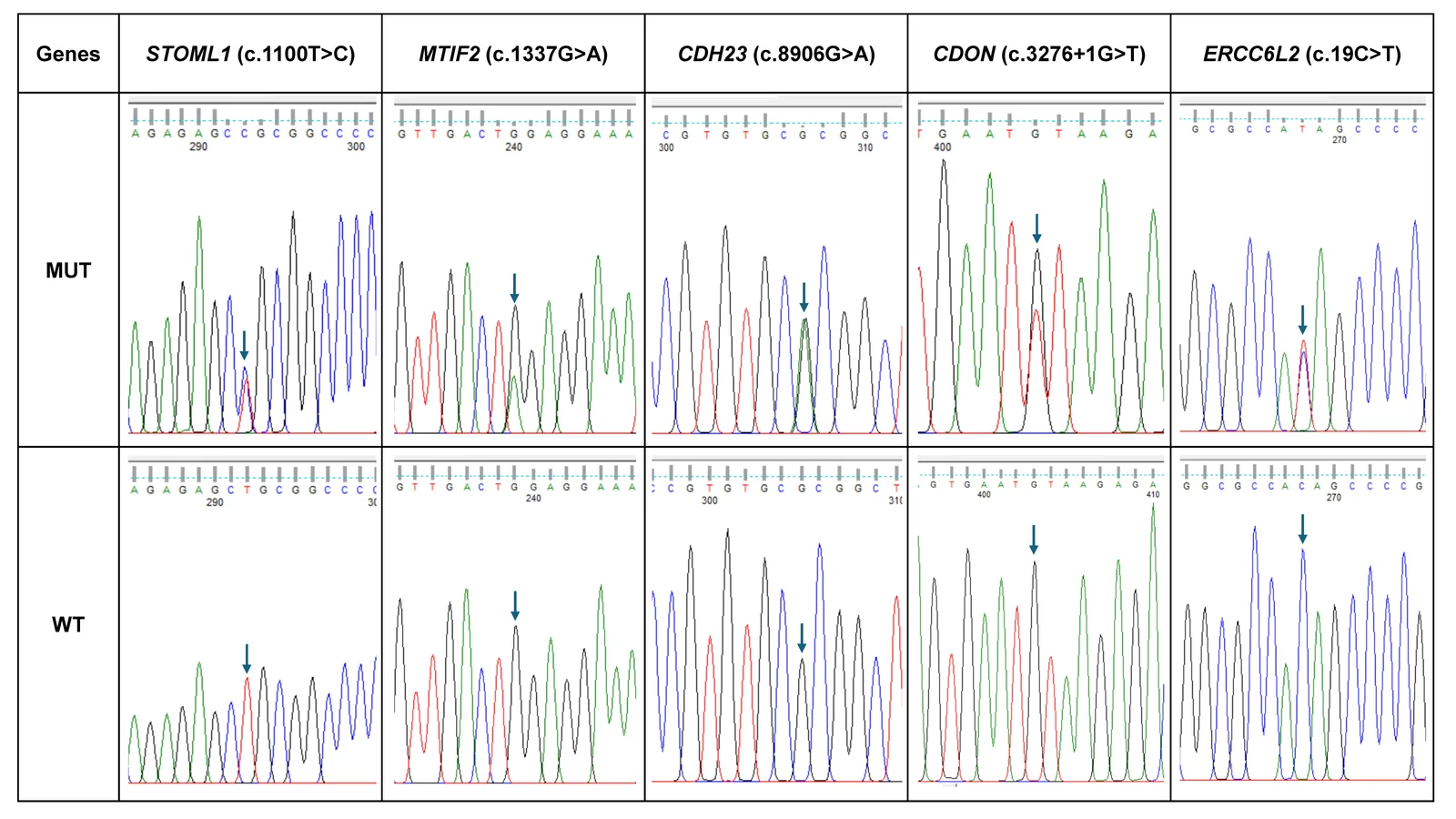
The validation of the five variants by Sanger sequencing. For each gene, the upper trace shows the heterozygous variant (MUT) and the lower trace the wild-type sequence (WT). The arrow indicates the variant.

To validate the variants identified by WES, direct Sanger sequencing was performed. Genomic DNA was amplified by PCR using GoTaq 2X master mix (Promega, Southampton, UK). Forward and reverse primers for *STOML1*, *MTIF2*, *CDH23*, *CDON*, and *ERCC6L2* were designed using Primer3 at https://primer3.ut.ee/ (accessed on 8 August 2025) (Table 2). PCR conditions were as follows: 94 °C for 5 min of initial denaturation, followed by 30 cycles of 30 s at 94 °C, 30 s at 60 °C, and 45 s at 72 °C. PCR products were purified and sequenced using BigDye Terminator v3.1 on an ABI 3730 Genetic Analyser (both Applied Biosystems, Foster City, CA, USA) and analysed using SeqMan Pro v8.0.2. Variants were identified in the four family members from whom DNA was available (I-2, III-6, IV-3, and IV-4); no DNA was available from any relative unaffected by coloboma other than IV-3 (Figure 3, Table 3). For this individual, the DNA sample was exhausted, precluding repeat Sanger sequencing or further segregation analysis. Therefore, no reliable genotype for the *CDH23* c.8906G>A variant was obtained.

### 2.4. Sequence Conservation Analysis

**Figure 4:**
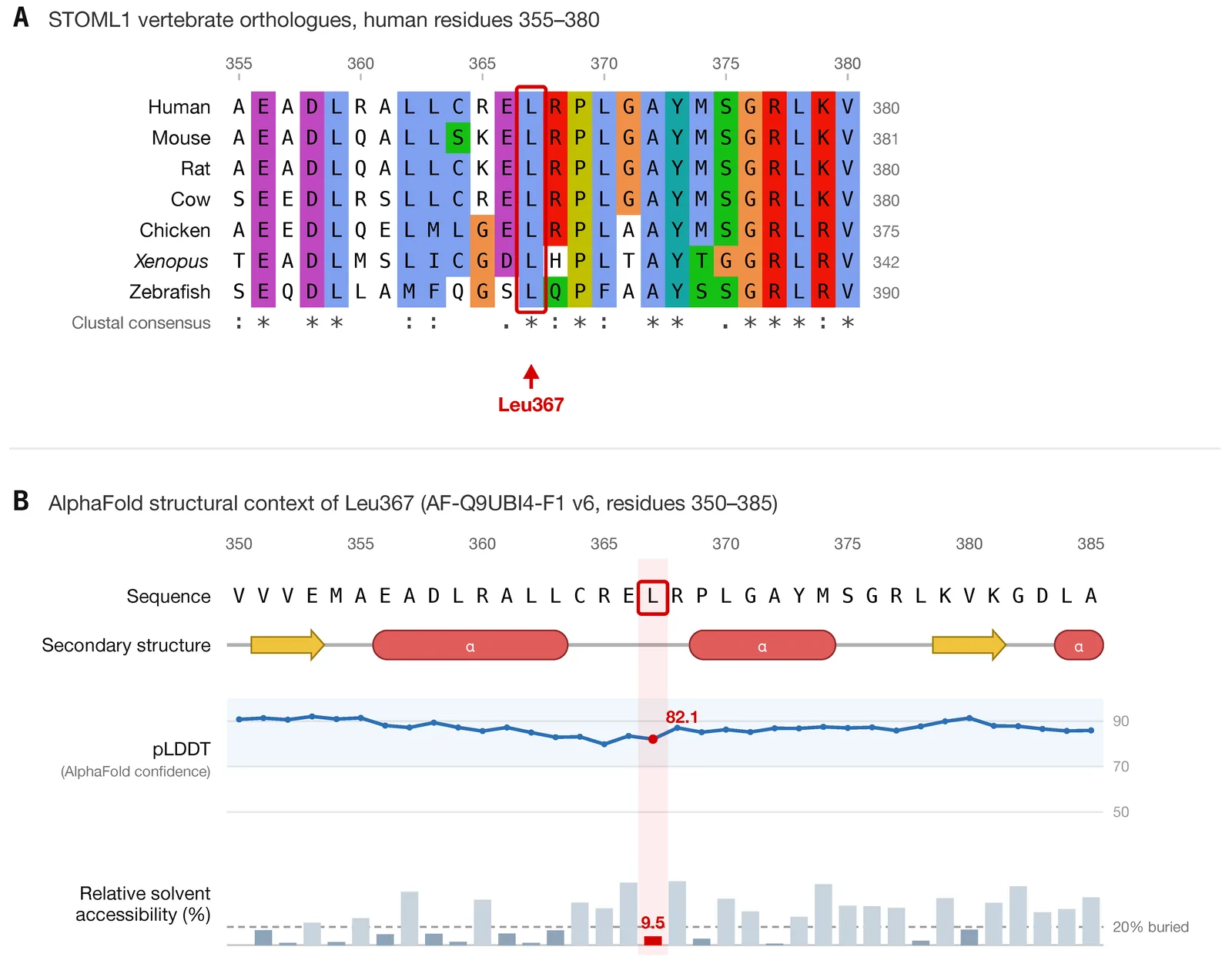
The conservation and structural context of STOML1 Leu367. (**A**) Alignment of STOML1 orthologues from seven vertebrate species over human residues 355–380; Leu367, indicated with arrow, is invariant. Residues are shaded using the ClustalX colour scheme. Clustal consensus symbols: * identical, : strongly similar, . weakly similar. (**B**) The structural context of Leu367 in the AlphaFold model of STOML1 (AF-Q9UBI4-F1 version 6) over residues 350–385, showing secondary structure, per-residue model confidence (pLDDT), and relative solvent accessibility. Leu367 lies in a short loop between two α-helices and is buried (relative solvent accessibility 9.5%).

STOML1 protein sequences were retrieved from UniProt and aligned using Clustal Omega version 1.2.4 at https://www.ebi.ac.uk/jdispatcher/msa/clustalo (accessed on 22 August 2026). Conservation at p.Leu367 was assessed across seven vertebrate orthologues spanning human to zebrafish (Figure 4A). The AlphaFold model for STOML1 (UniProt Q9UBI4; model AF-Q9UBI4-F1 version 6; https://alphafold.ebi.ac.uk (accessed on 22 August 2026)) was used to generate secondary structure, per-residue model confidence (pLDDT), and relative solvent accessibility at this position using the DSSP and Shrake–Rupley algorithms, respectively, and the predicted missense pathogenicity of p.(Leu367Pro) was taken from AlphaMissense. Anthropic Claude Opus 5 (https://claude.ai/; accessed on 22 August 2026) was used to assist in the production of this figure. All database accessions, sequence alignments, and derived values were checked against the source databases by the authors. The analysis scripts are available on request.

## 3. Results

A DNA sample from the proband (III-6), an affected individual, was submitted for WES. Five family members were clinically examined (I-2, II-5, III-6, IV-3, and IV-4). Interrogation of the available WES variant calls in known coloboma-associated genes identified no previously reported pathogenic SNV or small indel explaining the phenotype. Subsequent analysis using the Phenopolis pipeline identified rare candidate variants in three genes not previously implicated in coloboma (*STOML1*, *MTIF2*, *CDH23*) and one established coloboma gene (*CDON*), together with a variant in *ERCC6L2*, a gene associated with unrelated human disease. All identified variants are heterozygous (Table 1).

The four-generation family comprised seventeen members: eight affected, four unaffected (by coloboma; one unaffected individual IV-3 has paternal cataract), and five spouses. Five members of this family (I-2, II-5, III-6, IV-3, and IV-4) were examined at Moorfields Eye Hospital (Figure 2). One affected individual (III-6) underwent WES. Variant annotation and tiered filtering yielded five rare variants in *STOML1*, *MTIF2*, *CDH23*, *CDON*, and *ERCC6L2*. *CDON* is the only rare coding or splice-site variant identified in any of the coloboma-associated genes interrogated.

(i) A rare missense VUS in *STOML1* (NM_004809.5: c.1100T>C; p.(Leu367Pro); exon 7; chromosome 15q24.1).
(ii) A rare nonsense VUS in *MTIF2* (NM_002453.3: c.1337G>A; p.(Trp446Ter); exon 12; chromosome 2p16.1).
(iii) A rare missense VUS in *CDH23* (NM_022124.6: c.8906G>A; p.(Arg2969His); exon 61; chromosome 10q22.1).
(iv) A rare VUS in *CDON* (NM_001378964.1: c.3276+1G>T; p.?; intron 17; chromosome 11q24.2).
(v) A recurrent nonsense variant, classified as pathogenic in ClinVar, in *ERCC6L2* (NM_020207.7: c.19C>T; p.(Gln7Ter); exon 1; chromosome 9q22.32) (Table 1).

Sanger sequencing was performed in the four family members for whom DNA was available and confirmed the candidate variants where successful genotyping was possible (Figure 2, Table 3). The *STOML1* and *CDH23* variants were present in all three affected individuals tested (I-2, III-6 and IV-4). The *STOML1* variant was absent in IV-3, who has paternally inherited cataract but no coloboma. A reliable genotype for the *CDH23* variant could not be obtained for IV-3, and the remaining DNA from this individual was exhausted, preventing repeat analysis. Consequently, segregation data cannot distinguish definitively between *STOML1* and *CDH23*. The *MTIF2* variant was also present in IV-3 and therefore did not segregate cleanly with coloboma. The *CDON* variant was absent from the affected daughter IV-4, and the *ERCC6L2* variant was absent from the affected individual I-2; these variants were therefore excluded as the segregating cause of coloboma in this family.

## 4. Discussion

Currently, more than eighty genes have been implicated in MAC. The available WES variant dataset was interrogated for SNVs and small indels in known coloboma-associated genes, and no previously reported pathogenic variant of these classes explaining the phenotype was identified. In this four-generation family with isolated iris and chorioretinal coloboma, five rare heterozygous variants were prioritised (Table 1). Genotyping in the four family members from whom DNA was available excludes *CDON* and *ERCC6L2* as the segregating cause (Table 3). Taken with the mechanistic and in silico evidence discussed below, *STOML1* is the leading candidate and *CDH23* a weaker secondary candidate.

STOML1 (stomatin-like protein 1) is a band-7/SPFH-domain protein that localises to both the plasma membrane and the late endosomal compartment and acts as an important protein-lipid scaffold. It has been found to play a role in intracellular cholesterol trafficking, endosomal sorting [16], and the modulation of acid-sensing ion channels [17,18]. So far, no Mendelian disease has been associated with *STOML1*. Four family members were sequenced. In the three affected individuals, we identified a rare *STOML1* missense variant (p.(Leu367Pro)), which was absent in the one individual who did not have coloboma. This analysis does not establish a causal gene–disease relationship. Of the 126 rare coding or splice-site variants with CADD > 15 identified across the proband’s exome, approximately eight would be expected to show the same pattern of presence in the three genotyped affected individuals and absence in the genotyped unaffected individual by chance alone, taking the probability of that pattern as one in sixteen.

Leu367 is invariant across the seven vertebrate orthologues examined, from human to zebrafish (Figure 4A), and lies within the SCP-2 sterol-transfer domain of STOML1. In the AlphaFold model, the residue is predicted with strong confidence (pLDDT 82.1) and buried, lying in a short loop between two α-helices (Figure 4B). Thus, substitution by proline is predicted to disrupt local packing. AlphaMissense scores p.(Leu367Pro) at 0.888, classifying it as likely pathogenic. These observations are consistent with but do not establish, a deleterious effect. In the absence of functional data, this variant is classified as VUS and *STOML1* is put forward as a candidate gene requiring replication in other families.

Mitochondrial translational initiation factor 2 (MTIF2), a nuclear-encoded protein, is important in the initiation of protein synthesis within mitochondria. No validated gene-disease relationship has been established for *MTIF2* [19]. *MTIF2* variants have not been linked to ocular anomalies, although the retina and optic nerve are among the most metabolically active tissues, so they depend heavily on mitochondrial function for energy production. Systemic mitochondrial dysfunction thus frequently presents with ophthalmic features such as optic neuropathy or retinopathy [20,21,22].

*MTIF2* c.1337G>A; p.(Trp446Ter) causes a premature termination codon in exon 12, upstream of the last exon–exon junction, and is therefore predicted to trigger nonsense-related decay. Loss of the C-terminal region required for initiator tRNA binding and stable association with the mitochondrial small ribosomal subunit would therefore be expected to impair mitochondrial translation and oxidative phosphorylation. However, this variant was carried not only by the three affected individuals genotyped but also by IV-3, who has paternally inherited cataract and no coloboma. The variant therefore does not segregate cleanly with coloboma in this family and would require incomplete penetrance to remain tenable.

*CDH23* encodes cadherin-23, a member of the cadherin superfamily responsible in large part for governing cell–cell adhesion, tissue morphogenesis, and maintenance of tissue architecture [23]. Biallelic *CDH23* variants cause Usher syndrome type 1D and non-syndromic deafness DFNB12 [24]. In this family, the three affected individuals genotyped (I-2, III-6, and IV-4) harboured a rare missense variant (p.(Arg2969His)) in *CDH23*; no genotype was available for IV-3. However, it must be noted that the established disease mechanism for *CDH23* is recessive, whereas a single heterozygous change is reported here; *CDH23* is a large, missense-tolerant gene carrying many rare variants in population databases (gnomAD v4 allele frequency 2.11 × 10^−5^). Furthermore, AlphaMissense classifies this variant as likely benign (0.130), whereas *STOML1* p.(Leu367Pro) is classified as likely pathogenic (0.888) (Table 1). *CDH23* is therefore regarded as a weaker secondary candidate for coloboma in this family.

*CDON* (Cell Adhesion Associated, Oncogene Regulated) codes for a cell-surface receptor of the immunoglobulin superfamily, mediating interactions between muscle precursor cells and upregulating myogenesis. CDON is implicated in eye development, specifically in the formation and patterning of the optic vesicle and optic cup. Although it generally acts as a positive co-receptor, in the developing optic vesicle it instead functions as a decoy receptor for Sonic Hedgehog (Shh), influencing retinal development and optic nerve formation [25]. Variants in *CDON* can lead to holoprosencephaly and associated ocular abnormalities, including coloboma. Compound heterozygous splice variants in *CDON* (c.928+1G>A and c.2650+1G>T) are known to cause isolated coloboma [26]. We identified a rare splice-donor variant of uncertain significance in *CDON* (c.3276+1G>T; p.?) in the proband (III-6) only; it was absent in her affected daughter (IV-4), and no genotype was available for I-2. *CDON* cannot therefore account for the dominant trait in this family. We note that III-6 is the most severely affected member and is the sole carrier, which raises the possibility of a modifier effect; with a single carrier, however, this remains speculative.

*ERCC6L2* (Excision Repair 6 Like 2) encodes a Snf2-family helicase-like protein involved in DNA repair and mitochondrial function [27]. Biallelic variants in this gene cause bone marrow failure syndrome [28]. We identified the recurrent nonsense variant c.19C>T; p.(Gln7Ter) in III-6 and both of her daughters, but it was absent in the affected individual I-2, indicating that it entered the family from a different branch and does not segregate with coloboma. This variant has been previously documented in individuals with bone marrow failure. It is reported here as an incidental finding: the three carriers are heterozygous and are therefore not expected to be affected.

## 5. Conclusions

Here, we propose *STOML1* as an exploratory candidate gene in a four-generation British family with isolated iris and chorioretinal coloboma, wherein data were available for four individuals. Its presence in three affected individuals and absence in one unaffected individual are consistent with the phenotype but provide limited segregation evidence. *CDH23* remains a secondary candidate, while *CDON* and *ERCC6L2* are excluded as the segregating cause. Digenic inheritance has been proposed in coloboma for variants in *BEST1* and *RIMS1* [29], but the data presented here do not support a multi-genic model in this family.

This study has important limitations. We could not retrospectively assess the completeness of exon-level coverage across all known MAC-associated genes or perform additional read-depth-based CNV/structural-variant analysis. Pathogenic variants in poorly captured regions, exon-level deletions or duplications, larger structural variants, and non-coding regulatory regions therefore cannot be excluded. Further, DNA was available from only one relative unaffected by coloboma, so co-segregation could not be formally tested. The severity gradient observed across III-6, IV-4, and IV-3 correlates with the number of variants carried but is equally consistent with *STOML1* genotype alone. It is noteworthy, however, that the proband (III-6) developed severe cataract during pregnancy; thus, we hypothesise that there may be some broader oligogenic contribution outside of coloboma.

*STOML1* remains a promising exploratory candidate gene for iris and chorioretinal coloboma, inviting replication in independent families and functional validation in the future.

## Figures and Tables

**Table 1:** In silico pathogenicity predictions and classification for the five candidate variants.

GENES	HGVSC (GRCH37 COORDINATE)	HGVSP	GNOMAD V4 AF	GERP NR	CADD	DANN	ALPHA-MISSENSE	ACMG/AMP	CLASSIFICATION	CLINVAR
***STOML1***	c.1100T>C (chr15:74,276,375A>G)	p.(Leu367Pro)	8.21 × 10^−6^ (12)	4.28	26.0	0.9984	0.888 (likely pathogenic)	PM2_Supporting; PP3_Supporting	VUS	No data
***MTIF2***	c.1337G>A (chr2:55,470,779C>T)	p.(Trp446Ter)	6.84 × 10^−7^ (1)	5.46	46.0	0.9969	N/A (not missense)	PM2_Supporting; PVS1 not applicable	VUS	No data
***CDH23***	c.8906G>A (chr10:73,569,760G>A)	p.(Arg2969His)	2.11 × 10^−5^ (34)	5.28	27.5	0.9994	0.130 (likely benign)	PM2_Supporting; PP3 not met	VUS	No data
***CDON***	c.3276+1G>T (chr11:125,850,943C>A)	p.?	2.73 × 10^−4^ (441, 1 hom)	5.67	27.8	0.9958	N/A (not missense)	PM2 not met	VUS	VUS
***ERCC6L2***	c.19C>T (chr9:98,638,339C>T)	p.(Gln7Ter)	1.85 × 10^−4^ (293)	3.52	24.0	0.9690	N/A (not missense)	PVS1; PM2 not met	Recurrent/Pathogenic	Pathogenic

Allele frequencies are from the Genome Aggregation Database (gnomAD) v4.1.0, combining the exome and genome call sets with the allele count and the number of homozygotes given in brackets. Genomic Evolutionary Rate Profiling Neutral Rate (GERP NR) corresponds to the conservation score of the site. Combined Annotation Dependent Depletion (CADD) scores the deleteriousness of a variant; CADD score > 15 is considered damaging. AlphaMissense returns a variant-level pathogenicity score between 0 and 1 together with a benign, ambiguous, or likely pathogenic classification and is applicable only to missense variants. Deep Neural Network (DANN) is a score to predict the pathogenicity of the variant and ranges from 0 to 1; a higher value indicates that the variant is likely to be deleterious. ACMG/AMP criteria [15] are restricted to the population-frequency (PM2) and computational (PP3/BP4) evidence codes. AF, allele frequency; hom, homozygotes; VUS, variant of uncertain significance; N/A, not applicable. Variants are numbered on the reference transcripts *STOML1* NM_004809.5, *MTIF2* NM_002453.3, *CDH23* NM_022124.6, *CDON* NM_001378964.1, and *ERCC6L2* NM_020207.7; genomic coordinates are given on GRCh37/hg19.

**Table 2:** Primer sequences.

GENE	FORWARD PRIMER	REVERSE PRIMER
***STOML1***	ccaagagttcacacagccaat	GATGAACACCTCCTCCTCCA
***MTIF2***	caacacaaaggctttaatctagca	ggcccaaactatgagaataaaaa
***CDH23***	acttgcctgtcacctttgct	aaactgaggcccagagacct
***CDON***	tctcagcaagaagattgtccttt	caaagggctgccagtgat
***ERCC6L2***	GCCCGAGAGAACTAGGTGAA	tgaatcggagatttggttcc

Primers were designed using Primer3 at https://primer3.ut.ee/ (accessed on 8 August 2025).

**Table 3:** Genotypes of the five variants.

VARIANT	I-2	III-6 (PROBAND)	IV-4	IV-3	SEGREGATION WITH COLOBOMA
*STOML1* C.1100T>C	T/C	T/C	T/C	T/T	Consistent in all affected tested; absent in IV-3
*MTIF2* C.1337G>A	G/A	G/A	G/A	G/A	Not consistent—also carried by IV-3
*CDH23* C.8906G>A	G/A	G/A	G/A	No data	Indeterminate in all affected tested but IV-3 genotype unavailable
*CDON* C.3276+1G>T	No data	G/T	G/G	No data	Excluded—absent in affected IV-4
*ERCC6L2* C.19C>T	C/C	C/T	C/T	C/T	Excluded—absent in affected I-2

Patients I-2, III-6, and IV-4 are coloboma-affected; IV-3 has paternally inherited cataract without coloboma. Genotypes are given as reference/variant alleles, and all variants are heterozygous where present. No DNA were available from any relative unaffected by coloboma other than IV-3.

## Data Availability

The variant data discussed in this study are not readily available due to confidentiality protocols employed by the hospital from which the data is sourced. Requests to access these data should be directed to v.berry@ucl.ac.uk.
